# Control Room Operators’ Cue Utilization Predicts Cognitive Resource Consumption During Regular Operational Tasks

**DOI:** 10.3389/fpsyg.2019.01967

**Published:** 2019-08-27

**Authors:** Daniel Sturman, Mark W. Wiggins, Jaime C. Auton, Shayne Loft, William S. Helton, Johanna I. Westbrook, Jeffrey Braithwaite

**Affiliations:** ^1^Department of Psychology, Macquarie University, Sydney, NSW, Australia; ^2^School of Psychology, The University of Adelaide, Adelaide, SA, Australia; ^3^School of Psychological Science, The University of Western Australia, Perth, WA, Australia; ^4^Department of Psychology, George Mason University, Fairfax, VA, United States; ^5^Australian Institute of Health Innovation, Macquarie University, Sydney, NSW, Australia

**Keywords:** attentional processes, eye movements, near-infrared spectroscopy, process control, sustained attention

## Abstract

This study was designed to examine whether qualified practitioners’ cue utilization is predictive of their sustained attention performance during regular operational tasks. Simulated laboratory studies have demonstrated that cue utilization differentiates cognitive load during process control tasks. However, it was previously unclear whether similar results would be demonstrated with qualified practitioners during familiar operational tasks. Australian distribution network service provider (DNSP) operators were classified with either higher or lower cue utilization based on an assessment of cue utilization within the context of electrical power distribution. During two, 20-min periods of operators’ regular workdays, physiological measures of workload were assessed through changes in cerebral oxygenation in the prefrontal cortex compared to baseline, and through eye behavior metrics (fixation rates, saccade amplitude, and fixation dispersion). The results indicated that there were no statistically significant differences in eye behavior metrics, based on levels of cue utilization. However, as hypothesized, during both sessions, operators with higher cue utilization demonstrated smaller increases in cerebral oxygenation in the prefrontal cortex from baseline, compared to operators with lower cue utilization. The results are consistent with the proposition that operators with higher cue utilization experience lower cognitive load during periods of regular activity during their workday, compared to operators with lower cue utilization. Assessments of cue utilization could help identify operators who are better able to sustain attention during regular operational tasks, as well as those who may benefit from cue-based training interventions.

## Introduction

Control room operators in high risk industrial environments, including power and rail control, must respond rapidly and accurately to deviations in the system state (Stanton et al., 2009). These operators must visually search their systems for extended periods (Vicente et al., 2001). The failure to sustain attention may result in inaccurate or delayed responses, increasing the likelihood of potentially catastrophic errors (Reason, 2000).

Attentional resource theory posits that sustaining attention over extended periods results in the consumption of attentional resources (Kahneman, 1973; Helton and Russell, 2012). As attentional resources are limited, this can eventually result in fewer resources than are necessary to identify and manage changes in the system state (Parasuraman, 1979; Wickens, 2002). Consequently, a vigilance decrement is often observed during sustained attention tasks, whereby performance efficiency declines, as evident in increased response latency, and/or decreased accuracy in response to critical signals (Parasuraman, 1979; Helton et al., 2007b).

A resource depletion account of the vigilance decrement is supported by more cognitively demanding tasks resulting in steeper declines in performance (Helton and Russell, 2013; Shaw et al., 2013). For example, decreasing signal saliency or adding a secondary task results in increases in response latency and a greater frequency of missed critical signals (Helton and Warm, 2008). Conversely, reducing task demands, by increasing signal salience or by inserting warning signals, improves performance (MacLean et al., 2009).

Operators who experience less cognitive load during operational tasks should consume fewer resources over time (Kahneman, 1973). Consequently, these operators should retain greater residual cognitive resources, thereby enabling them to sustain their performance for longer periods (Matthews et al., 2010). Cue utilization is one strategy that operators utilize to reduce the rate at which cognitive resources are consumed (Wiggins, 2011).

### Cue Utilization

Cues are associations between situation-specific environmental features and task-related objects or events (Brunswik, 1955). Cue utilization is the application of cue-based processing, which is dependent upon individuals’ capacity to develop, and recognize cues (Lansdale et al., 2010). Cue utilization requires the identification of predictive features in the operational environment, the association between these features and events in memory, the retention of these cue-based associations, and the application of cues in response to environmental features (Wiggins, 2012).

The activation and retrieval of cues from long-term memory has the advantage of enabling performance while imposing relatively fewer demands on working memory resources (Chung and Byrne, 2008). Further, effective cue utilization should enable operators to attend to features of greater relevance, reducing the overall number of features to which they attend, thereby reducing the rate at which cognitive resources are consumed (Weiss and Shanteau, 2003; Sturman et al., 2019). For example, electricity network controllers may learn that certain patterns of failed circuit breakers are associated with specific system failures, enabling them to attend non-consciously to areas of the network where a fault is likely to have occurred, thereby increasing the likelihood of a rapid response, while minimizing workload.

Evidence to support the assertion that higher cue utilization is associated with the consumption of fewer cognitive resources can be drawn from research demonstrating that, during sustained attention tasks, participants with higher cue utilization report relatively lower perceived cognitive load, and record relatively smaller increases in response latency, compared to participants with lower cue utilization (Brouwers et al., 2016). Using a simulated rail control task containing implicit patterns of train movements, Brouwers et al. (2016) noted that the addition of a concurrent, secondary task negatively impacted the performance of participants with lower cue utilization, but had no impact on performance for participants with higher cue utilization. Participants with higher cue utilization presumably recognized the implicit pattern, which enabled the adoption of a cue-based strategy. This reduced the rate at which their cognitive resources were consumed, thereby providing additional residual resources that minimized the impact of the secondary task.

Although the effects appear relatively consistent, evidence to support the association between cue utilization, and the rate of cognitive resource consumption relies primarily on inferences derived from response latencies (Brunswik, 1955; Small et al., 2014; Brouwers et al., 2016, 2017). However, this association could potentially be explained by alternative factors, such as participants’ level of motivation or engagement. Further, assessments of cue utilization and sustained attention performance both typically rely on measures of response latency and accuracy, and data for each participant is typically collected in a single session (e.g., Brouwers et al., 2016, 2017). Consequently, the relationship between cue utilization and cognitive resource consumption may be partially attributable to common method bias. To overcome these potential methodological issues, complementary evidence is required using alternative measures of cognitive resource consumption.

Physiological measures of cognitive load, such as eye behavior metrics and near infrared spectroscopy (NIRS) enable the assessment of cognitive load during sustained attention tasks without the need for subjective ratings or performance measures (Poole and Ball, 2006; Helton et al., 2010). For instance, NIRS enables changes in cognitive load to be inferred through changes in cerebral oxygenation in the prefrontal cortex (Fishburn et al., 2014). Sustained attention tasks are typically associated with increased activity in the right prefrontal cortex (Helton et al., 2007a; Warm et al., 2009), with greater increases in cerebral oxygenation indicative of greater cognitive load (Fallgatter and Strik, 1997; Helton et al., 2010).

Sturman et al. (2019) examined the relationship between cue utilization and the consumption of cognitive resources amongst novice train control operators using eye tracking data to assess the frequency of fixations (fixation rates), and NIRS to measure cerebral oxygenation in the right prefrontal cortex. During novel rail control simulations containing repetitious patterns of train movement, participants with higher cue utilization (assessed in the domain of driving) demonstrated greater decreases in fixation rates and smaller increases in cerebral oxygenation in the prefrontal cortex, while maintaining a higher level of performance, compared to participants with lower cue utilization (Sturman et al., 2019). This evidence provides additional support for the proposition that higher cue utilization is associated with the consumption of fewer cognitive resources during novel sustained attention tasks. However, Sturman et al. relied on cross-task cue utilization, whereby cue utilization evaluated in one context (driving) was used to predict cognitive load in another novel context (rail control). As these novice operators had no prior opportunities to acquire relevant cues, differences in cognitive load based on cue utilization likely reflect differences in the rate of cue acquisition during the novel tasks. Consequently, it remains unclear whether differences in cognitive load based on cue utilization are also evident amongst qualified personnel in familiar operating environments.

In complex operating environments, the emergence of critical features is likely to be less predictable compared to experimental tasks containing repetitious patterns. While fixation rates provide an indication of search pattern efficiencies in predictable environments, additional eye behavior metrics may be required to provide measures of search patterns in less predictable environments. For example, researchers often analyze a range of eye behavior metrics, including saccade amplitude and fixation dispersion, to examine hazard detection during dynamic driving tasks (Underwood et al., 2011).

Saccade amplitude and fixation dispersion can be used to assess workload and the consumption of cognitive resources during sustained attention tasks (Di Nocera et al., 2007). Saccade amplitude refers to the change in the degrees of visual angle from the pre-saccade fixation to post-saccade fixation. Greater performance in the detection of critical targets has been associated with search strategies involving saccades of smaller amplitude (Bertram et al., 2013). Fixation dispersion is the extent to which fixations are distributed while completing a task. Smaller fixation dispersions have been associated with lower subjective ratings of workload during simulated flight (Di Nocera et al., 2007) and during visuomotor tasks (Camilli et al., 2007).

The aim of the present study was to examine whether qualified operators’ cue utilization is associated with cognitive resource consumption during regular operational tasks. To assess cue utilization and cognitive resource consumption in an operational context, operators from four Australian distribution network service provider (DNSP) control rooms, responsible for remotely monitoring, and controlling electrical power distribution networks, were recruited to participate in the study. Cue utilization, assessed in the context of electrical power control, was used to predict physiological measures of cognitive load during periods of operators’ regular workdays. As the activation and retrieval of cues from long-term memory has the advantage of imposing relatively fewer demands on working memory resources, operators with higher cue utilization should consume fewer cognitive resources during regular operational tasks. Further, based on the proposition that operators with higher cue utilization will draw on patterns in memory to anticipate events and enable more efficient search patterns, these operators should spend more time attending to specific areas associated with their operational tasks, rather than broadly scanning their environment. Specifically, it was hypothesized that, during periods of their regular workday, control room operators with higher cue utilization would record lower increases in cerebral oxygenation from baseline, lower visual fixation rates, smaller mean fixation dispersions, and smaller mean saccade amplitudes, compared to operators with lower cue utilization.

## Materials and Methods

### Design

Testing sessions were conducted during two, 20-min periods of each participant’s regular workday. The two sessions were not necessarily comparable, as the interval between the first and second sessions varied. Consequently, the two sessions were analyzed separately. Each session comprised a 2 × 4 mixed-methods factorial design incorporating two levels of cue utilization (higher and lower) as a between-subjects factor, and four, 5-min time periods (Periods 1–4) as a within-subjects factor. Time constituted the four quartiles of the 20-min testing sessions. Participants were classified with either higher or lower cue utilization based on an assessment of cue utilization within the context of power distribution.

### Participants

Participants comprised 38 male distribution power controllers, recruited from four Australian DNSP control rooms. The sex distribution in this case reflects the fact that this tends to be a male dominated industrial workplace. Participants ranged in age from 27 to 60 years (*M* = 42.2, *SD* = 7.6), had acquired a mean 8.8 years (*SD* = 4.8) of experience as network controllers, and had acquired a mean 19.9 years (*SD* = 9.7) working in power distribution.

### EXPERTise 2.0

EXPERT intensive skills evaluation (EXPERTise 2.0) is an on-line assessment tool designed to assess behavior consistent with the utilization of cues (Wiggins et al., 2015). For the current study, EXPERTise 2.0 was tailored to the domain of power distribution. Tasks in the EXPERTise battery include a feature identification task (FIT), a feature recognition task (FRT), a feature association task (FAT), a feature discrimination task (FDT), and a feature prioritization task (FPT).

In the feature identification task, participants are presented with a series of domain-related stimuli and are asked to identify, as quickly as possible, the key feature of concern. In the distributed network service provider (DNSP) edition of EXPERTise, six scenarios were provided, each of which consisted of an electrical line diagram representing a transformer failure, a voltage under or overload, or a normal condition within a substation. The substations were generic to account for the different display systems that are employed by the different participating organizations, with participants offered the opportunity to become familiar with the substations prior to commencing the FIT. For each scenario, participants were asked to select the area of most concern within the substation line diagram. In the FIT, response latency is measured as the time in milliseconds from the initial presentation of the stimulus to the selection of an area of concern. Higher cue utilization is associated with shorter mean response latencies (Loveday et al., 2013).

For the feature recognition task, participants are asked to categorize complex, domain-related stimuli that are presented for a short period. The intention is to assess the capacity of participants to rapidly extract key information to form accurate classifications. In the DNSP edition of EXPERTise 2.0, participants were presented with 10 line diagrams, similar to those used in the FIT. Each diagram was displayed for between 20 and 60 s depending upon the complexity of the scenario. Following the removal of the line diagram, participants were asked to select, from one of five options, the condition represented in the preceding display (e.g., “The substation has suffered a loss of all indications”). In this case, higher cue utilization is associated with a greater number of correct classifications (Wiggins and O’Hare, 2003; Brouwers et al., 2017).

Participants completing the FAT are presented with two domain-related stimuli, either simultaneously or sequentially, and are asked to rate their perceived relatedness on a six-point scale ranging from 1 (*Extremely unrelated*) to 6 (*Extremely related*). For the DNSP edition of EXPERTise 2.0, participants were presented with 13 pairs of phrases simultaneously that comprised features and associated objects/events related to power distribution (e.g., “overhead lines” and “low voltage”). Each pair of phrases was presented for 2 s, after which participants were asked to rate the perceived relatedness of the two phrases. Higher cue utilization is associated with a greater mean variance in the perceived relatedness of terms against the mean response latency (Morrison et al., 2013).

In the feature discrimination task, participants are presented with a problem-oriented scenario, and are asked to formulate an initial response from a list of possible responses. Subsequently, participants are presented with a list of features described in the scenario, and are asked to rate each of the features based on their perceived relevance to the initial response, using a 10-point scale ranging from 1 (*Not important at all*) to 10 (*Extremely important*). The DNSP edition of EXPERTise consisted of two, detailed power distribution scenarios (e.g., hot air balloon caught in overhead wires). In the FDT, higher cue utilization is associated with a greater variance in ratings (Weiss and Shanteau, 2003; Pauley et al., 2009).

The feature prioritization task is a time-limited task in which participants are provided with a brief statement that frames a domain-related problem. It is intended to assess participants’ capacity to prioritize the acquisition of information during problem orientation. Accompanying the statement is a series of buttons that are listed vertically and labeled as features related to the problem. Selecting a button provides information that further elucidates the nature of the problem and provides the basis for a response. In the DNSP edition of EXPERTise 2.0, two scenarios were included (e.g., received a notification from the call center that a member of the public has lodged an emergency call), where the acquisition of information was limited to 90 s. Lower cue utilization is associated with the selection of information in the sequence in which it is presented (e.g., from top to bottom of the display), while higher cue utilization is associated with a lower frequency of menus selected in the sequential order in which they are presented (Wiggins and O’Hare, 1995; Wiggins et al., 2002).

### Eye Tracking

Prior to each *in situ* testing session, participants were fitted with SMI eye tracking glasses (version 2) using the system’s standard operating procedures, including a three-point calibration. Eye tracking data were recorded for the duration of each 20-min testing session. Fixation rates, fixation dispersion, and saccade amplitude were calculated for each of the four time periods throughout each testing session. Fixation rates were calculated as the mean frequency of eye fixations recorded per minute. Saccade amplitude was calculated as the mean change in degrees of the visual angle per saccade, while fixation dispersion was calculated as the mean dispersion of fixations in degrees of visual angle. Multiple fixations and saccades were not recorded during head movements or during smooth pursuit (SensoMotoric Instruments, Teltow, Germany).

Due to calibration difficulties, eye behavior metrics were unable to be accurately calculated for two participants during Session 1, one in the high cue utilization typology and one in the low cue utilization typology, and for three participants during Session 2, one in the high cue utilization typology and two in the low cue utilization typology. Consequently, data for these participants were excluded from the respective analyses involving fixation rates, saccade amplitude, and fixation dispersion.

### Near Infrared Spectroscopy (NIRS)

Prior to each *in situ* testing session, participants were fitted with a Portalite NIRS sensor (Portalite, Artinis Medical Solutions, Netherlands), which uses light in the near-infrared spectrum to measure cerebral activation. The Portalite NIRS system consists of three channels, with inter-optode distances of 30, 35, and 40 mm, enabling a penetration depth of 20 mm into the prefrontal cortex. Consistent with previous research, the sensor was positioned approximately one centimeter above the participants’ right eyebrow, to enable measurement of cerebral oxygenation in the right prefrontal cortex, while avoiding sinus cavities and hair that might interfere with the signal (Yoshitani et al., 2002; Helton et al., 2007a). As oxyhemoglobin (O2Hb) and deoxyhemoglobin (HHb) have distinct optical absorption characteristics, NIRS can be used to determine the relative amounts of each in the cerebral tissue. The ratio of O2Hb to total hemoglobin (O2Hb + HHb) is used to calculate regional oxygen saturation (rSO2), which can be used as a measure of cerebral activation (Ekkekakis, 2009).

The baseline period was 2 min prior to each testing session, during which time participants were asked to sit quietly, minimize body movements and to remain as relaxed as possible. rSO2 during the second minute of the baseline period was used as a baseline index. rSO2 scores for each of the four, 5-min work periods were calculated by comparing mean rSO2 during each period of the *in situ* testing to the baseline rSO2 measure. Scores represent the percentage change in rSO2 from baseline, with positive scores representing an increase in rSO2 compared to baseline, and negative scores represented a decrease in rSO2 compared to baseline.

### Subjective Workload

Subjective workload was measured using a version of the NASA Task Load Index (NASA-TLX: Hart and Staveland, 1988). The NASA-TLX is a tool that records subjective perceptions of workload along the six dimensions of physical demands, mental demands, temporal demands, effort, frustration, and performance. Twice during each testing session, participants were asked to rate their perception of workload during the preceding 10 min, using a seven-point Likert scale for each dimension of workload.

NASA-TLX scores were used to establish the work demands perceived by operators during each testing session, with higher scores indicating a busier period of work. As work demands were not able to be controlled experimentally during field testing, subjective workload scores enabled perceived work demands to be controlled statistically. Consequently, any differences in cognitive load (as measured by rSO2) observed during statistical analyses reflect expected differences in cerebral activation independent of perceived task demands.

### Procedure

This research complied with the American Psychological Association code of ethics and was approved by the Institutional Review Board at Macquarie University. Informed consent was obtained from each participant. Testing consisted of an online component and an *in situ* component. For the online component, power controllers from the four DNSP control rooms were provided general information and given the URL to the EXPERTise 2.0 website. Participants then answered a series of questions that were incorporated to generate a unique participant code. Participants provided their age, sex, the number of years that they had been employed as a network controller, and the number of years they had worked in power distribution. On completion of the demographic questions, the participants completed EXPERTise.

During the *in situ* testing sessions, participants were invited to wear eye-tracking glasses and a near-infrared spectroscope during two, 20-min periods of their regular workday, once near the beginning of their shift and once toward the end of their shift. After participants gave their consent, they answered the same series of questions used to generate their unique participant code in the online component, allowing their data to be matched anonymously. Participants were then fitted with the eye-tracking glasses and near-infrared spectroscope, and instructed to continue with their current work tasks as they would during a typical workday. Participants were asked to complete the NASA-TLX twice during each testing session.

During each testing session, network operators’ primary task was to monitor a supervisory control and data acquisition (SCADA) interface consisting of an operating power distribution grid, to make routine adjustments when necessary, and to respond appropriately to any deviations in the network. The SCADA interfaces consisted of three or four monitors arranged in a standardized arrangement around each operators’ work station. While operators did not monitor the same section of the power distribution grid during testing sessions, their substantive roles and responsibilities were consistent.

## Results

### Physiological Measures

Mean fixation dispersion and saccade amplitude were log transformed to correct for positively skewed distributions. Fixation rates and rSO2 scores were approximately normally distributed for each period and cue utilization typology.

### Cue Utilization Typologies

EXPERTise data were used to identify cue utilization typologies that corresponded to higher or lower levels of cue utilization. Consistent with a standard approach for classifying participants into cue utilization typologies (Wiggins et al., 2014), scores for each task were converted to *z*-scores, and a cluster analysis was used to identify two typologies. The first cluster, labeled the higher cue utilization typology, consisted of participants, the centroids for whom reflected a shorter response latency on the FIT, greater accuracy on the FRT, a higher mean ratio of variance to reaction time on the FAT, a greater variance in ratings on the FDT, and a higher mean ratio of sequential selections in the FPT. The second cluster, labeled the lower cue utilization typology, consisted of participants, the centroids for whom reflected the opposite pattern to the higher cue utilization typology. There were significant differences in FIT, FRT, and FDT mean scores between the higher and lower cue utilization groups (see Table 1). In the case of the FDT and FPT, the differences were non-significant. Nevertheless, the pattern of responses was generally consistent with the pattern which would normally be expected to characterize higher or lower cue utilization.

**TABLE 1:** Cluster centroids for the EXPERTise task scores.

**EXPERTise**	**Cluster 1 (*n* = 14)**	**Cluster 2 (*n* = 24)**		
**Tasks**	**Higher cue utilization**	**Lower cue utilization**	***t***	***p***
FIT	−0.52	0.31	2.66^∗^	0.012
FRT	0.58	−0.34	3.00^∗∗^	0.005
FAT	0.64	−0.37	3.44^∗∗^	0.001
FDT	0.20	−0.12	0.94	0.338
FPT	0.39	−0.23	1.87	0.117

*^∗^Significant at the 0.05 level (two-tailed). ^∗∗^Significant at the 0.01 level (two-tailed); df = 36.*

### Covariates

#### Power Distribution Experience

Independent samples *t*-tests indicated that there was no statistically significant difference in the number of years in which operators had been employed as network controllers for the lower cue utilization typology (*M* = 9.2, *SD* = 5.2), compared to participants allocated to the higher cue utilization typology (*M* = 8.1, *SD* = 4.1), *t*(36) = 0.70, *p* = 0.487, *d* = 0.24. Further, there was no statistically significant difference in the number of years that operators had worked in power distribution for the lower cue utilization typology (*M* = 22.2, *SD* = 10.2), compared to participants in the higher cue utilization typology (*M* = 16.0, *SD* = 7.5), *t*(36) = 1.98, *p* = 0.056, *d* = 0.60. Pearson’s correlations indicated that years as a network controller was positively correlated with mean fixation rate during Session 1 (*r* = 0.406, *p* = 0.014) and Session 2 (*r* = 0.372, *p* = 0.028), and negatively correlated with fixation dispersion during Session 1 (*r* = −0.340, *p* = 0.043) and Session 2 (*r* = −0.362, *p* = 0.032), and was therefore included as a covariate for analyses involving eye behavior metrics. No other statistically significant correlations were evident between years of experience and rSO2 levels or saccade amplitude (*p*s > 0.05).

#### Subjective Workload

Independent samples *t*-tests indicated that cue utilization was not associated with any dimension of the NASA-TLX (*p*s > 0.05). Pearson’s correlations for Session 1 revealed a statistically significant, positive association between rSO2 levels and temporal demands, *r* = 0.320, *p* = 0.023, and between rSO2 levels and effort, *r* = 0.323, *p* = 0.022. Pearson’s correlations for Session 2 revealed a statistically significant positive association between rSO2 levels and mental demands, *r* = 0.350, *p* = 0.014, and between rSO2 levels and effort, *r* = 0.375, *p* = 0.008. Other correlations between the dimensions of subjective workload and the outcome variables during Sessions 1 and 2 were not statistically significant (*p*s > 0.05). Consequently, mental demands, temporal demands, and effort were included as covariates for the main analyses involving rSO2.

### Cue Utilization and rSO2

**FIGURE 1:**
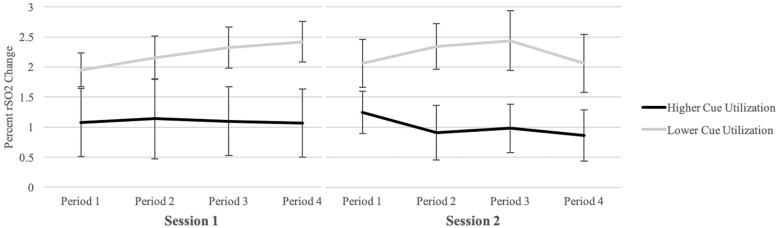
Mean oxygenation scores in the right hemisphere by cue utilization typology and time during Session 1 **(left)** and Session 2 **(right)**. Oxygenation scores are based on percent change relative to baseline. Error bars represent ±1 SE.

Independent samples *t*-tests indicated that there were no statistically significant differences in baseline rSO2 based on cue utilization for Session 1 (*p* = 0.735), or Session 2 (*p* = 0.601). A 2 × 4 ANCOVA was conducted for each session, with cue utilization as a between-groups variable (higher and lower), time as a within-groups variable (Periods 1–4), mental demands, temporal demands, and effort as covariates, and rSO2 scores as the dependent variable (see Figure 1). For Session 1, a statistically significant main effect was evident for cue utilization, *F*(1,33) = 5.21, *p* = 0.029, η^2^ = 0.136, with the lower cue utilization typology recording significantly greater increases in rSO2 from baseline (*M* = 2.21, *SD* = 1.28) compared to the higher cue utilization typology (*M* = 1.10, *SD* = 1.69). There was no statistically significant main effect for time, *F*(3,99) = 1.21, *p* = 0.309, η^2^ = 0.035, and no statistically significant interaction between cue utilization and time, *F*(3,99) = 1.07, *p* = 0.366, η^2^ = 0.031.

For Session 2, a statistically significant main effect was evident for cue utilization, *F*(1,33) = 4.65, *p* = 0.038, η^2^ = 0.123, with the lower cue utilization typology recording significantly greater increases in rSO2 from baseline (*M* = 2.23, *SD* = 2.05) compared to the higher cue utilization typology (*M* = 0.95, *SD* = 1.43). There was no statistically significant main effect for time, *F*(1.8,60.1) = 1.96, *p* = 0.145, η^2^ = 0.056, and no statistically significant interaction between cue utilization and time, *F*(1.8,60.1) = 1.24, *p* = 0.298, η^2^ = 0.036.

### Cue Utilization and Eye Behavior Metrics

A 2 × 4 MANCOVA was conducted for each session, with cue utilization group as a between-groups variable (higher and lower), time as a within-groups variable (Periods 1–4), and years of experience as a network controller as a covariate. The dependent variables comprised fixation rates, saccade amplitude, and fixation dispersion.

The multivariate effect was not statistically significant by cue utilization during Session 1, *F*(1,33) = 0.46, *p* = 0.509, η^2^ = 0.013, nor during Session 2, *F*(1,32) = 0.02, *p* = 0.884, η^2^ = 0.001. There was no statistically significant multivariate effect of time during Session 1, *Wilk’s Lambda* = 0.42, *F*(3,31) = 0.46, *p* = 0.715, η^2^ = 0.042, nor during Session 2, *Wilk’s Lambda* = 0.99, *F*(3,30) = 0.08, *p* = 0.968, η^2^ = 0.008. No statistically significant multivariate interaction was evident between time and cue utilization during Session 1, *Wilk’s Lambda* = 0.85, *F*(3,31) = 1.85, *p* = 0.158, η^2^ = 0.152, nor during Session 2, *Wilk’s Lambda* = 0.85, *F*(3,30) = 1.72, *p* = 0.184, η^2^ = 0.147. This indicates that, during both Sessions, there were no differences in eye behavior metrics based on either cue utilization or time.

## Discussion

The primary aim of this study was to examine whether qualified operators’ cue utilization is associated with the consumption of cognitive resources during regular operational tasks. DNSP system operators were classified with higher or lower cue utilization typologies within the context of power distribution. During two, 20-min periods of operators’ regular workdays, physiological measures of workload were assessed through changes in cerebral oxygenation in the prefrontal cortex, and through eye behavior metrics.

As higher cue utilization is associated with the identification of more predictive features and greater efficiencies in information processing (Lansdale et al., 2010; Wiggins, 2015), greater cue utilization should be associated with more efficient search patterns, and the consumption of fewer cognitive resources. Consequently, it was hypothesized that operators with higher cue utilization would record lower increases in cerebral oxygenation from baseline, lower visual fixation rates, smaller mean fixation dispersions, and smaller mean saccade amplitudes, compared to operators with lower cue utilization.

The results indicated that there were no statistically significant differences in eye behavior metrics, based on levels of cue utilization. However, as hypothesized, during both Sessions, operators with higher cue utilization demonstrated smaller increases in cerebral oxygenation in the prefrontal cortex from baseline, compared to operators with lower cue utilization. Higher cue utilization amongst operators is associated with less cognitive resource expenditure during operational tasks.

### Theoretical and Practical Implications

The outcomes of the present study are consistent with previous research, demonstrating that higher cue utilization is associated with smaller increases in cerebral oxygenation in the prefrontal cortex (Sturman et al., 2019). However, tasks in these previous experiments consisted of laboratory simulations, which contained predicable feature-event relationships, compared to the real operational environment used in the current study to which experts are exposed. Consequently, it was unclear whether context-based cue utilization would predict cognitive load amongst qualified operators during regular operational tasks where the emergence of critical features was likely to be less predictable compared to artificial laboratory tasks containing highly repetitious patterns.

The present study extends previous research, providing support for the proposition that qualified operators’ cue utilization predicts cognitive resource consumption during operational tasks. Further, differences in cerebral oxygenation were evident, controlling for participants’ subjective ratings of workload. This suggests that, when completing tasks that are similarly demanding, operators with lower cue utilization are likely to consume a greater proportion of their cognitive resources over a specified time period, compared to operators with higher cue utilization. As a reduction in the availability of cognitive resources is associated with an increase in operational errors (Wickens, 1980; Reason, 1990), operators with lower cue utilization are more likely to demonstrate a greater decline in performance during operational tasks.

Greater residual cognitive resources are also posited to allow operators with higher cue utilization to better manage additional task demands (Brouwers et al., 2017). Consequently, assessments of cue utilization may aid in the selection of job applicants who are better able to sustain attention and maintain performance during more demanding situations. Further, assessments of cue utilization could be used to aid the training and professional development of operators. For instance, the ability to predict the rate at which operators consume cognitive resources could be used to improve job performance by optimizing the length of time between breaks for individual operators. Alternatively, assessments of cue utilization could help identify operators who would benefit from cue-based training interventions, whereby operators are given the opportunity to acquire cues that can be generalized to the broader operational environment (Wiggins, 2015).

### Limitations and Future Direction

The present study is limited by the lack of experimental control which occurs during field testing. For instance, due to time constraints and operators’ work schedules, testing sessions for different operators were conducted at different times of the day and inevitably during periods of relatively higher or lower work demands. While work demands during testing sessions may have differed between participants, there is no evidence suggesting a systematic variation in work demands between the higher and lower cue utilization typologies. Nevertheless, to help control for variances in work demands, two sessions were conducted for each operator during different periods of their shift. Further, subjective self-reports of workload were collected to control statistically for differences in perceived work demands. This point notwithstanding, a strong feature of the current work is the ecological validity afforded by testing expert operators performing real operational power control tasks.

### Conclusion

The current study was designed to determine whether qualified operators’ cue utilization is associated with cognitive resource consumption during regular operational tasks. Physiological measures of cognitive resource consumption were assessed during two, 20-min periods of power distribution network controllers’ regular work days. During both sessions of testing, operators with higher cue utilization demonstrated smaller increases in cerebral oxygenation in the prefrontal cortex from baseline, compared to operators with lower cue utilization. However, cue utilization was not associated with differences in eye behavior. The results of the study are consistent with the proposition that power operators with higher cue utilization are likely to consume cognitive resources at a slower rate during regular operational tasks, compared to operators with lower cue utilization.

## Data Availability

All datasets generated for this study are included in the manuscript and/or the supplementary files.

## Ethics Statement

This study was carried out in accordance with the recommendations of the National Statement on Ethical Conduct in Human Research (2007) and the Macquarie University Faculty of Human Sciences Human Research Ethics Sub-Committee with written informed consent from all subjects. All subjects gave written informed consent in accordance with the Declaration of Helsinki. The protocol was approved by the Macquarie University Faculty of Human Sciences Human Research Ethics Sub-Committee.

## Author Contributions

All authors conceived and designed the study, and wrote the manuscript. DS, MW, and JA collected the data. DS and MW analyzed and interpreted the data.

## Conflict of Interest Statement

The authors declare that the research was conducted in the absence of any commercial or financial relationships that could be construed as a potential conflict of interest.
